# Supplemental education in early childhood may be associated with professional achievement

**DOI:** 10.5116/ijme.5cda.79ab

**Published:** 2019-05-24

**Authors:** Teruyuki Kajiume, Masao Kobayashi

**Affiliations:** 1 Mukainada Child Clinic, Hiroshima, Japan, and Department of Pediatrics, Graduate School of Biomedical Sciences, Hiroshima University, Hiroshima, Japan; 2 Department of Pediatrics, Graduate School of Biomedical Sciences, Hiroshima University, Hiroshima, Japan

**Keywords:** Education, medical doctors and students, extracurricular activities, keyboard lessons, pre-school children

## Abstract

**Objectives:**

To investigate which extracurricular lessons medical doctors and medical students received in early childhood and to compare the results to a similarly aged representative sample.

**Methods:**

This retrospective questionnaire-based study investigated the prevalence of supplemental early education, along with professional outcomes later in life. The study compared two samples: First, as a proxy for “professional success”, medical students and residents (n = 147) were asked to recall which extracurricular lessons they had taken when pre-school aged. This was contrasted to a control sample representative of the general population in Japan. Included extracurricular lessons were: “keyboard/piano”, “Japanese calligraphy”, “abacus use”, “swimming”, and “foreign language.” Frequencies were compared and tested using contingency tables and the Chi-squared test. P-values < 0.05 were considered significant.

**Results:**

The control sample reported a lower rate (32.7%) of extracurricular activities than medical students did (51.6%, χ^2^_(df=1, n=147) _= 13.5, p < 0.001). The proportion of medical students receiving keyboard lessons during their pre-school years was significantly higher (43.5%) than that of the general population (9.1%, χ^2^_(df=1, n=147)_ = 65.2, p < 0.001). Similar, but less robust, results were observed with Japanese calligraphy (11.5% vs. 3.1%, χ^2^_(df=1, n=147)_=11.3, p=0.001), abacus use (4.1% vs. 0.4%, χ^2^_(df=1, n=147)_ = 7.4, p=0.007), and swimming (33.3% vs. 22.0%, χ^2^_(df=1, n=147) _= 6.1, p = 0.013).

**Conclusions:**

Our results suggest that, in Japan, early supplementary education, including keyboard lessons, is associated with professional success later in life. Future research is warranted to elucidate whether there is a causal link between early extracurricular education and professional outcomes.

## Introduction

It has been previously reported that the environment in which a child is reared is strongly associated with academic achievement in adulthood.^1^ Specifically, Matsushige^1^ showed a strong correlation between early elementary school achievement and later educational achievement and high-status occupations. Additionally, children of parents with advanced degrees, such as medical degrees, were more likely to themselves attain advanced degrees and prestigious occupations.^1^

A child’s school achievement may be shaped by several factors, including genetic predisposition, parents’ commitment to education, and socioeconomic status. Additionally, the teacher-child relationship plays a significant role in child development. Past studies investigating children from kindergarten through fifth grade have indicated that increasing the quality of the teacher-child relationship leads to improvements in academic achievement.^2^ As such, eventual occupational choice, and, by extension economic and social stability, is likely to be partially determined early, during children’s elementary-school years.

Supplemental lessons are a way for parents to provide early stimulation for children above and beyond what is provided by the (elementary) school. Previous reports stress that early music training enhances children’s reading and writing skills.^3^^,^^4^ In fact, children receiving musical training displayed structural brain changes associated with improvement in motor and auditory skills, highlighting that practicing a skill such as music early in life results in training-induced brain activity.^5^ Furthermore, physical activity such as extracurricular sports may also improve academic outcomes, at least in the short-term.^6^

Factors other than education influencing academic success include genetic predisposition. Identical twins raised in different environments were found to have similar intelligence quotient (IQ) levels.^7^ However, the relationship between intelligence and genetics is not absolute, and environmental factors influence IQ. The heritability of IQ is highest in affluent families, whereas in families with low socioeconomic status the environment is a larger determinant.^8^ The IQ of medical doctors is among the highest of all professions,^9^ and while genetics are important, it is likely that such individuals’ parents were also committed to thorough education. In this study, we investigated which types of extracurricular education medical residents and medical students received as children through a questionnaire-based survey. This group was chosen as an indirect proxy of intelligence and professional success. The main aim was to identify whether these participants, medical students, and residents, were more likely to have received supplemental education in addition to regular schooling in their childhood. Results are contrasted to a sample consisting of participants reflective of the general population.

We hypothesize that lessons in addition to regular education in early life are associated with, and may influence, future professional success. This study contributes to the understanding of which childhood activities may be most associated with later academic achievement and professional success.

## Methods

### Study population

This questionnaire-based study included 85 medical students in their 5th and 6th year of training and 62 medical residents (total n=147) from the Mukainada Child Clinic at Hiroshima Hospital. Participants were recruited between September 2010 and June 2011 and included all new medical students and residents from the department. The respondents’ ages ranged from 22 to 29 years; 62 were female, and 85 were male. There were no exclusion criteria in this study. Participants were asked to recall the types of lessons they had received in addition to their regular schooling when they were between the ages of 3 and 6, using a structured questionnaire. These included lessons in: “keyboard/piano,” “Japanese calligraphy,” “abacus use,” “swimming,” and “foreign language.” Participants were informed of the purpose of the investigation and provided written informed consent. The study was approved by the Ethical Committee of Hiroshima University Hospital in August of 2018.

### Comparative analysis

In order to facilitate comparative analysis, our data on residents and medical students were contrasted to control data representative of the general population. This comparative dataset was obtained from Video Research Ltd. (Tokyo, Japan), market research and survey corporation. The video research consisted of data from 1994 (the “investigation of children” study), coinciding with the timeframe when our sample of medical students were children. In addition, a second and similar dataset of video research with recent data (2010, the “characters and children market investigation” study) was obtained to investigate whether extracurricular activity patterns have changed over a generation. In April 1994, 600 school aged children aged 3–12 years (excluding junior high-school students) who lived in a 30-km radius surrounding Tokyo Station were recruited by Video Research Ltd. Data was obtained via interview during home visits. Researchers explained the purposes of the study and obtained consent. In total, 254 pre-school children (age 3–6) were included, and their data were used for comparative analysis in the current study. For reliability analysis (1994 vs. 2010), we included similar survey data from June 2010, where, as of April 1994, 618 children (n=249 pre-school children) who lived in a 30-km radius surrounding Tokyo Station were recruited. This 2010 sample was also extracted from the Video Research monitor list and was similar in sex ratio and age composition the 1994 group.

### Statistical analysis

Frequencies were analyzed using the Chi-squared test and Fisher’s exact test, where appropriate. Chi-squared values are reported and p-values less than 0.05 were considered statistically significant.

## Results

**Figure 1:**
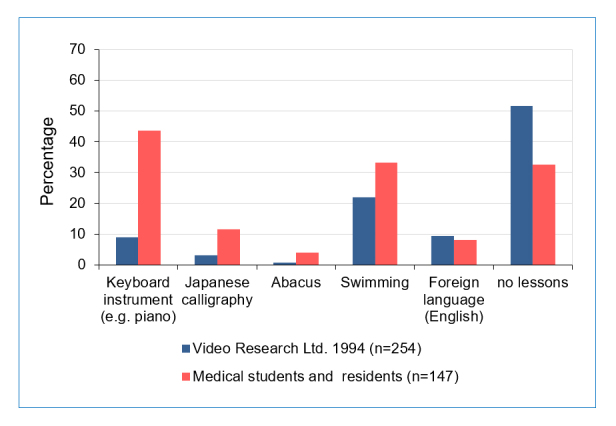
Comparisons of the ratio of lessons during the pre-school period of the representative control sample (1994) with the questionnaire-based survey of medical students and residents

Medical students and residents were compared with control data from Video Research Ltd. obtained in 1994 when both groups were in their pre-school years (Figure 1). The ratio of medical students and residents who did not take any extracurricular lessons during the pre-school period was significantly lower compared to the control sample (32.7% vs 51.6%, χ^2^_(df=1, n=147)_ =13.5, p<0.001). Interestingly, the ratio of specific supplementary lessons differed considerably depending on activity. Keyboard lessons (e.g., the piano) were significantly more likely to be taken by medical students and residents compared to the control sample (43.5% vs. 9.1%, χ^2^_(df=1, n=147) _= 65.2, p<0.001). Additionally, the ratio of classes in Japanese calligraphy (11.5% vs. 3.1%, χ^2^_(df=1, n=147)_=11.3, p=0.001) and abacus use (4.1% vs. 0.4%, χ^2^_(df=1, n=147)_=7.4, p=0.007) were significantly higher, as was the ratio of swimming (33.3% vs. 22.0%, χ^2^_(df=1, n=147)_=6.1, p= 0.013). However, the percentage of children learning foreign languages (e.g. English) did not differ between the two groups (8.2% vs. 9.4%, χ^2^_(df=1, n=147)_ = 0.2 p=0.664).

**Figure 2:**
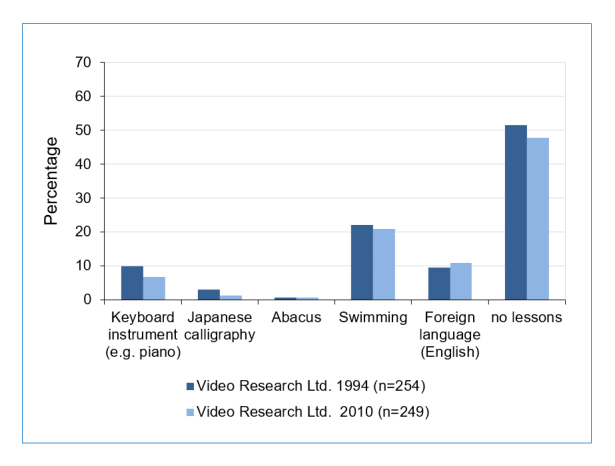
Comparisons of the ratio of lessons during the pre-school periods of 1994 and 2010

In order to determine whether the frequency of extracurricular lessons may have changed over almost two decades, control data from 1994 were compared to a newer control sample obtained in 2010. The ratio of participants taking

particular lessons during their pre-school years in 1994 and 2010 are shown in Figure 2. In these two representative samples of the general population, the prevalence of none of the lessons changed significantly between 1994 and 2010 (χ^2^_(df=1, n=147)_ <3.8, p>0.05), suggesting that lesson habits have not changed over time.

## Discussion

In this study, we aimed to investigate whether early-life supplemental lessons were more prevalent among children who became successful professionally later in life than among a sample representative of the general population. We show that the children becoming medical students and residents in their twenties had when they were pre-school aged, significantly higher rates of keyboard/piano lessons in particular. In addition, they were more likely to participate in lessons in calligraphy, abacus, and swimming, but not a foreign language. As a secondary outcome, we compared whether there was a difference in the ratio of supplemental lessons received by control children in 1994 and 2010. We report no such differences, highlighting that in representative samples of Japanese children studied almost 20-years apart, the generational change did not affect the frequency of supplemental lessons.

We recruited 147 medical students and residents from the Mukainada Child Clinic of Hiroshima Hospital. Participants were asked to recall the following childhood extracurricular activities: “keyboard instrument,” “Japanese calligraphy,” “abacus use,” “swimming,” and “foreign language.” Additional responses (painting, football, judo, gym, table tennis, Japanese dance, violin, ballet, and marimba) were obtained but were not included for analysis in the present study. Comparative analysis was not possible regarding these items, as they were not included in the data from Video Research Ltd. For the comparative analysis, a total of 254 control participants were extracted from Video Research Ltd. data. These participants were part of an ongoing market research study and were included in the present study as they were the same age (pre-school age around 1994 when the survey data was collected) as our sample of medical students and residents. A secondary cohort from Video Research Ltd. was added (2010 cohort) to compare the ratio of extracurricular lessons across generations.

Even though many factors, including genetic and socioeconomic factors, play an important role in a child’s achievement, it has been shown that early education and teacher involvement has a large impact on future economic and social stability.^2^ As parents have minimal control over schooling within the classroom, some may choose to supplement with after-school lessons. Such activities can range from music and academic lessons to increased physical activity, all of which have shown some potential for improving academic outcomes.^3^^-^^6^ For example, a previous study demonstrated the positive impact of direct and strenuous physical movement and a negative impact of inactive time on wellbeing and academic accomplishment.^10^

Differences between study groups were the largest regarding keyboard lessons. Medical students and residents were substantially more likely to take such lessons compared to the general population. Music is among the most perplexing of human phenomena and is practiced in some form and researched worldwide.^11^ Studies have investigated the cognitive effects of music on audiences and its therapeutic application for patients.^12^^-^^15^ In 1993, Rauscher et al. reported that, after listening to a Mozart sonata, students’ mean spatial IQ scores were higher than those measured in a state of relaxation and silence. As a result, educational activities using music by Mozart became popular.^11^ However, other groups have not been able to reproduce these findings.^16^ A recent study using Mozart and retrograde Mozart music with both humans and rats as models revealed that subjecting participants to Mozart had positive cognitive effects, while the retrograde Mozart music had a negative effect on cognition in both human and rat populations.^17^ In our study, we did not directly measure intelligence. However, by using professional success as a marker of intelligence, one could argue that there is a correlation between music lessons and intelligence. Causality can, however, not be determined and should be investigated in future studies. Previous reports stated that music lessons might improve learning ability itself.^18^ In addition, there have been reports that IQ can increase after keyboard and voice lessons when compared to drama or no lessons. Taken together with our findings, this suggests that keyboard lessons may be a potential target for extracurricular activities.^19^

The one negative finding from this study relates to lessons in a foreign language. Most often this language is English, and the odds of a child receiving such lessons was not significantly higher (or lower) in the medical group. The official language of Japan is Japanese, and few Japanese individuals speak other languages. In Japan, people are rarely in contact with English-speaking populations. It is a common observation that most of the Japanese population are not proficient in the English language despite studying it for more than six years in school. The number of children learning foreign languages in Japan has not increased over time. Even though these observations do not explain our results, it is interesting to note that taking English lessons as a child is evidently not a deterministic factor of future success, and although speculative, it suggests that Japanese parents may not view English as a purely academic pursuit with the goal of increasing a child’s academic abilities.

Based on previous research and our current findings, we can speculate that extracurricular lessons positively influence academic performance. However, one of the main limitations of the present study is that due to study design we cannot conclusively establish whether keyboard lessons, calligraphy, abacus use, and swimming have a causal relationship with later academic performance and brain development. Moreover, in this study, we contrasted two separate datasets which, although comparable, potentially open the door to bias as a result of underlying differences in, for example, recruitment procedures and in-person interviews with children vs. recall in adults. Another limitation is that we used a sample of medical students and residents from a single Japanese pediatrics department as a proxy for professional success later in life. As such, our findings may not be fully generalizable to other measures of adult success or other, non-Japanese, cultures.

It is conceivable that our results reflect a positive feedback loop where successful and high economic-status parents encourage their children to participate in lessons in addition to regular schooling, while these lessons, in turn, improve academic and professional outcomes. We observed an especially strong difference in the prevalence of keyboard or piano lessons and professional success in later life, and, although we cannot establish a causal effect, the results from this study may be beneficial to parents who wish to enroll their children in lessons in addition to regular education in schools

## Acknowledgments

We are grateful to Video Research Ltd. for providing the standard data. We thank the medical students and residents who answered questionnaires. Editorial support in the form of medical writing, assembling tables and creating high-resolution images based on authors’ detailed directions, collating author comments, copyediting, fact-checking, and referencing was provided by Editage, Cactus Communications. We did not receive any funding for this research

## Conflict of Interest

The authors declare that they have no conflict of interest.
